# The Inflation Reduction Act and Patient Costs for Drugs to Treat Heart Failure

**DOI:** 10.1001/jamanetworkopen.2024.41915

**Published:** 2024-10-29

**Authors:** Erin Trish, Karen Van Nuys, Joanne Wu, Nihar R. Desai

**Affiliations:** 1 Leonard D. Schaeffer Center for Health Policy and Economics, University of Southern California, Los Angeles; 2 Department of Pharmaceutical and Health Economics, Mann School of Pharmacy and Pharmaceutical Sciences, University of Southern California, Los Angeles; 3 Yale School of Medicine, New Haven, Connecticut

## Abstract

This cross-sectional study examines the pre–Inflation Reduction Act out-of-pocket burden on Medicare patients taking dapagliflozin, empagliflozin, and sacubitril/valsartan.

## Introduction

The 2022 Inflation Reduction Act (IRA) contains several provisions to lower Medicare drug costs, including permitting the Centers for Medicare & Medicaid Services (CMS) to limit the prices of certain medicines and altering the standard Part D benefit to limit patient out-of-pocket costs.^1^ CMS has set the prices of 10 drugs effective 2026, including 3 commonly prescribed as part of combination therapy for heart failure (HF): dapagliflozin, empagliflozin, and sacubitril/valsartan.^2^ Dapagliflozin and empagliflozin also treat other conditions, including diabetes and chronic kidney disease.

One goal of the IRA is to reduce patient out-of-pocket liability; previous research has shown that the out-of-pocket cap will benefit patients taking HF drugs.^3^ However, the effect of eliminating the coverage gap phase has gotten less attention. Although previous legislation technically closed the coverage gap, patients still often faced mid-year out-of-pocket increases on branded drugs, with some reporting discontinuing their medication due to difficulty affording their prescriptions.^4,5^ Our study examines the pre-IRA out-of-pocket burden on Medicare patients taking these 3 drugs.

## Methods

This cross-sectional study was reviewed by the University of Southern California institutional review board and determined to be exempt from informed consent because it met the criteria for coded private information or biological specimens. This study followed the Strengthening the Reporting of Observational Studies in Epidemiology (STROBE) reporting guideline. We analyzed 100% Medicare prescription claims data from 2021, including stand-alone Part D plans and those offered with common Medicare Advantage plans (eg, HMOs, PPOs). We excluded claims for beneficiaries enrolled in employer and special needs plans and for beneficiaries under age 65 years and those who qualified for low-income subsidies. Each claim records the patient’s out-of-pocket cost separately from the plan cost. Claims were standardized to 30-day supply equivalents and grouped by benefit phase (deductible, pre-initial coverage limit [pre-ICL], ICL [ie, coverage gap], and catastrophic). Claims straddling multiple phases were categorized by their ending phase. We tested differences in median out-of-pocket costs across benefit phases using a Kruskal-Wallis test. A 2-sided *P* < .05 was considered significant. Data were analyzed from March to August 2024. Statistical analyses were conducted using SAS Enterprise Guide Version 7.15HF9 (SAS Institute).

## Results

Across 630 448 individuals with at least 1 claim for any of the 3 drugs (mean [SD] age, 73.6 [6.3] years; 396 573 males [62.9%]; 233 875 females [37.1%]), total out-of-pocket spending was $244 296 641 (empagliflozin: $122 843 585; sacubitril/valsartan: $76 347 630; dapagliflozin: $45 105 426) (Table). The median (25-75 percentiles) out-of-pocket costs per 30-day supply in the ICL phase were roughly 3 times those in the pre-ICL phase (empagliflozin: $144 [$131-$146] vs $46 [$40-$47]; dapagliflozin: $141 [$130-$142] vs $47 [$40-$47]; sacubitril/valsartan: $153 [$140-$155] vs $47 [$42-$47]; all differences significant at *P* < .001). Out-of-pocket costs were the lowest in the catastrophic phase.

**Table.** Medicare Out-of-Pocket Spending by Benefit Phase for Dapagliflozin, Empagliflozin, and Sacubitril/Valsartan (2021)^a^

Drug name	Benefit phase of the Part D event				All
	Deductible	Pre-ICL	ICL	Catastrophic	
**Sacubitril/valsartan**					
No. of claims	1718	370 632	326 535	110 890	809 775
Out-of-pocket spending per 30-d standardized claim, $					
Median (IQR)	563 (307-617)	47 (42-47)	153 (140-155)	31 (30-31)	47 (42-153)
Mean (SD)	444 (210)	71 (88)	138 (38)	40 (30)	94 (78)
Sum, $^b^	762 515	26 194 940	44 958 476	4 431 699	76 347 630
**Dapagliflozin**					
No. of claims	314	226 369	196 307	95 529	518 519
Out-of-pocket spending per 30-d standardized claim, $					
Median (IQR)	564 (513-569)	47 (40-47)	141 (130-142)	28 (28-28)	47 (35-141)
Mean (SD)	461 (198)	74 (89)	127 (37)	34 (25)	87 (73)
Sum, $^b^	144 840	16 813 147	24 866 764	3 280 675	45 105 426
**Empagliflozin**					
No. of claims	974	651 226	555 087	243 202	1 450 489
Out-of-pocket spending per 30-d standardized claim, $					
Median (IQR)	571 (47-581)	46 (40-47)	144 (131-146)	29 (28-29)	47 (37-144)
Mean (SD)	383 (249)	66 (80)	128 (39)	36 (26)	85 (70)
Sum, $^b^	372 801	42 708 777	70 951 915	8 810 092	122 843 585

Abbreviation: ICL, initial coverage limit.

^a^ Data are presented for out-of-pocket spending per 30-day standardized claim. Claims that straddle multiple benefit phases are included in the benefit phase in which they end. For example, if a claim straddles ICL and catastrophic phases, it is counted as a catastrophic claim. Median out-of-pocket spending per 30-day standardized claim was significantly different among benefit phases for each drug, all *P* < .001.

^b^ Total out-of-pocket spending on these drugs that occurred in that benefit phase.

Pre-ICL claims peak in January at 200 159 (97.7% of monthly claims) (Figure). ICL claims increased steadily to 136 397 (55.6%) in August; they accounted for more than one-third of monthly claims in each month from May through December. Catastrophic claims grew steadily throughout the year, peaking at 107 098 (40.1%) in December. Very few claims occured in the deductible phase.

**Figure.**
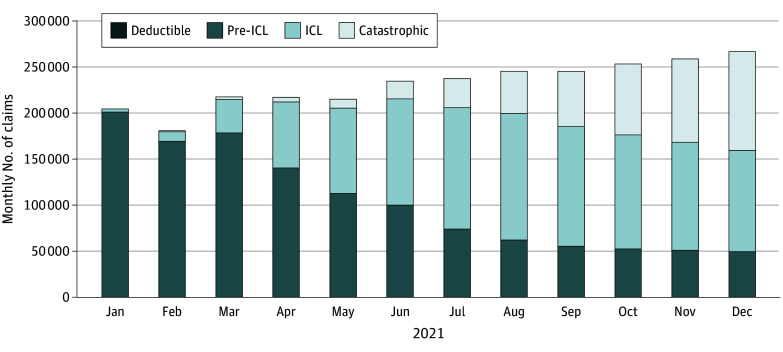
Monthly Count of Claims for Dapagliflozin, Empagliflozin, and Sacubitril/Valsartan by Benefit Phase (2021) Claims that straddle multiple benefit phases are included in the benefit phase in which they end. For example, if a claim straddles initial coverage limit (ICL) and catastrophic phases, it is counted as a catastrophic claim. Each bar represents the number of claims for the 3 study drugs falling in that month, stratified by benefit phase. The number of claims varied by month (January, 204 921; February, 181 025; March, 217 491; April, 217 187; May, 215 275; June, 234 838; July, 237 818; August, 245 462; September, 245 390; October, 253 469; November, 259 041; December, 266 867).

## Discussion

In this cross-sectional study of Medicare beneficiary costs for 3 IRA-targeted drugs, median out-of-pocket burden peaked in the coverage gap phase, where most June to December claims occurred. As the IRA’s standard benefit redesign eliminates the coverage gap in 2025, and caps annual out-of-pocket expenditures, it will reduce and smooth patient out-of-pocket burden. It is unclear what additional benefit the IRA’s price setting provision will provide. How these IRA provisions will impact access, adherence, and clinical outcomes remains unknown but represents an important area for further study. Study limitations include potential changes in out-of-pocket spending since 2021.

## Notes

Supplement.
Data Sharing Statement
