# Supplementary material for: The Inflation Reduction Act and Patient Costs for Drugs to Treat Heart Failure
Source: JAMA Netw Open. 2024 Oct 29;7(10):e2441915. doi: 10.1001/jamanetworkopen.2024.41915 (PMC11522936; doi:10.1001/jamanetworkopen.2024.41915)
Supplement: Supplement. — Data Sharing Statement [file jamanetwopen-e2441915-s001.pdf]

Data Sharing Statement

Data
Data available: No

Additional Information
Explanation for why data not available: We cannot provide access to Medicare claims subject to our data use agreement with CMS. We can provide instructions on how individuals can establish a DUA with CMS and details on data construction and analysis permissible within the terms of our DUA.
